# Serum miR‐205‐5p level for non–small‐cell lung cancer diagnosis

**DOI:** 10.1111/1759-7714.14356

**Published:** 2022-02-17

**Authors:** Li‐Guo Lu, Guo‐Ming Zhang

**Affiliations:** ^1^ Shuyang Hospital Shuyang Jiangsu China; ^2^ The Affiliated Shuyang Hospital of Xuzhou Medical University Shuyang Jiangsu China

**Keywords:** diagnosis, miR‐205‐5p, non–small‐cell lung cancer

Zhao et al.^1^ reported “MiR‐205‐5p promotes lung cancer progression and is valuable for the diagnosis of lung cancer” in an article published in *Thoracic Cancer*. To evaluate the diagnostic efficiency of serum miR‐205‐5p for lung cancer (LC), the investigators collected 75 LC patients and 62 healthy volunteers in this study. Determination of serum miR‐205‐5p using quantitative polymerase chain reaction (qPCR) and the diagnostic performance of serum miR‐205‐5p level by the area under the curve (AUC) of a receiver operating characteristic (ROC) curve analysis to discriminate non–small‐cell lung cancer (NSCLC) patients from the healthy population. These researchers found that serum miR‐205‐5p level was significantly higher in NSCLC than healthy controls, and the AUC is 0.8250. Therefore, these researchers concluded that the serum miR‐205‐5p was a novel diagnostic biomarker for NSCLC.

The study by Zhao et al.^1^ is very interesting for discovering a novel diagnostic biomarker for LC. However, the paper has some limitations. First, the number of LC patients is more than the controls, and the controls have only healthy controls without LC. To our knowledge, the controls include benign diseases and healthy population in cancer research, and the number of the cancerous subjects is less than the number of the control. The research belongs to case–control study in this article.

Second, the research was not enrolled in a study cohort consecutively from the pre‐designed inclusion. The design is not a one‐gate design study.^2^ The LC patients and the controls are from a cohort. The number of LC patients and controls are constant and fixed. Therefore, the sensitivity and specificity of diagnostic tests will be also constant.^2^ In the research, the cancerous subjects and the controls do not come from the same cohorts. The number of the cancerous subjects is crucial, if the number is higher, there will be higher‐estimated sensitivity. Whereas the number of controls is higher, there will be more and more estimate of specificity.^2^ Therefore, the specificity and sensitivity of this research is strongly affected by the controls.^3^, ^4^ The controls (including benign disease and healthy population) are very crucial for evaluating serum miR‐205‐5p level for LC diagnosis. Healthy populations do not have any risk factors, symptoms, signs related to LC patients, and it is not necessary to determine serum miR‐205‐5p to distinguish between healthy population and LC patients. It should be using one‐gate design in this research based on using both pre‐specified inclusion and exclusion criteria for consecutively enrolling. In this research, including LC patients, benign lung diseases, and healthy controls whose serum miR‐205‐5p should be tested.

In conclusion, this research seems to provide serum miR‐205‐5p as a novel diagnostic biomarker for NSCLC. However, they would be better to use a one‐gate design to obtain a well‐designed study because of the limitations of this study. As we know, the controls should include healthy population and benign diseases, and the number of cancerous subjects is less than the number of controls.

## CONFLICT OF INTEREST

The authors of this letter declare no conflicts of interest.

## Data Availability

The raw data supporting the conclusions of this manuscript will be made available by the authors, without undue reservation, to any qualified researcher.
